# Carriage rates and antimicrobial resistance of *Staphylococcus aureus* and methicillin-resistant *Staphylococcus aureus* in Ethiopia: A systematic review and meta-analysis

**DOI:** 10.1371/journal.pone.0333054

**Published:** 2025-10-10

**Authors:** Mengistie Yirsaw Gobezie, Minimize Hassen, Tewodros Solomon, Mulat Belete Demessie, Getahun Worku, Husien Nurahmed Toleha, Nuhamin Alemayehu Tesfaye

**Affiliations:** 1 Department of Clinical Pharmacy, College of Medicine and Health Sciences, Wollo University, Dessie, Ethiopia; 2 Department of Statistics, College of Natural Sciences, Wollo University, Dessie, Ethiopia; 3 Department of Social and Administrative Pharmacy, College of Medicine and Health Sciences, Wollo University, Dessie, Ethiopia; Children's Hospital at Westmead, AUSTRALIA

## Abstract

**Background:**

*Staphylococcus aureus* (*S. aureus*) and methicillin-resistant *S. aureus* (MRSA) are significant public health concerns globally due to their high prevalence and increasing antimicrobial resistance. This study aimed to assess the carriage rate of *S. aureus* and its antimicrobial resistance profile in Ethiopia.

**Methods:**

A comprehensive literature search was conducted in electronic databases, including PubMed, Google Scholar, Hinari, SCOPUS, and EMBASE, for articles published between 2013 and 2024. From 159 potential studies, 19 observational studies reporting *S. aureus* carriage rates and/or antimicrobial susceptibility in Ethiopia were included in the final analysis. The Joanna Briggs Institute (JBI) critical appraisal checklist was used to assess the quality of the studies. The data synthesis and reporting were conducted in accordance with the PRISMA checklist guidelines. A random-effects meta-analysis was performed to pool the estimates. Heterogeneity was assessed using Q statistics, I^2^ statistics, meta-regression, and sensitivity analysis. Publication bias was evaluated subjectively with a funnel plot and Egger’s regression test, with p < 0.05 indicating the presence of publication bias.

**Results:**

The pooled carriage rate of S. aureus was 25% (95% CI: 21–29%). The pooled odds ratio between MRSA and MSSA was 0.31 (95% CI: 0.21–0.46). Subgroup analysis showed the highest carriage rates before 2019, in the Oromia and SNNPR regions and HIV-positive patients. Egger’s regression test (p < 0.001) and trim and fill analysis adjusted the pooled carriage rate to 17.4% (95% CI: 12.6–22.2). Sensitivity analysis confirmed the robustness of the findings. High resistance rates were observed for penicillin (93%), ampicillin (80%), and amoxicillin (67%), with vancomycin (3%), ceftriaxone (5%), and clindamycin (6%) showing the lowest resistance rates.

**Conclusion:**

This systematic review and meta-analysis revealed a substantial carriage rate of *S. aureus* in Ethiopia, with nearly one-third of the isolates being MRSA. Significant regional and demographic variations were observed. These results underscore significant regional variations, highlighting the need for targeted interventions. High resistance rates to common antibiotics were found, emphasizing the need for enhanced infection control and judicious antimicrobial use in Ethiopia.

## Introduction

Antimicrobial drug resistance has emerged as a global threat and continues to pose a significant challenge to medicine and healthcare systems worldwide [1]. The use of antibiotics can lead to microbial resistance, which poses significant challenges to the prevention and management of infectious diseases [2]. *S. aureus*, a Gram-positive coccous bacterium, is found in the skin, intestines, nasal passages, and throat. It causes a wide range of infections in humans, from minor skin and soft tissue infections to life-threatening systemic disorders [3,4]. The term MRSA specifies the variants of *S. aureus* that are resistant to medications like oxacillin, methicillin, cephalosporins and/or nafcillin [2]. Globally, the prevalence of *S. aureus* has quickly increased. The majority of countries reported having more than 20% and sometimes even up to 80% of MRSA [5]. Notably, the nasal epithelium has the highest colonization rate, with an average prevalence of 40% in the adult population [6].

In addition to the prevalence of *S. aureus* nasal carriage rate, the prevalence of MRSA carriage rate is increased rapidly throughout the world. Methicillin-resistant *Staphylococcus aureus* (MRSA) is resistant to methicillin and other beta-lactam antibiotics due to the presence of the mecA gene, which encodes an altered penicillin-binding protein (PBP2a) with low affinity for these drugs. In contrast, methicillin-sensitive *Staphylococcus aureus (*MSSA) lacks the mecA gene and relies on its normal PBPs for cell wall synthesis, which are effectively inhibited by methicillin [7–9]. Multidrug-resistant MRSA is widespread in the majority of Asian hospitals; estimates range from 28% (in Hong Kong and Indonesia) to >70% (in Korea) [10]. Additionally, it is estimated that of all isolated *S. aureus* strains in Saudi Arabia, 25% had nasal carriage of MRSA. [2] The prevalence rate of MRSA strains in Africa is likewise comparatively high, ranging from 21% to 30%, with the majority (> 60%) of these strains being multiresistant [11]. Although the studies included in the review primarily involved hospitalized patients, which does not independently represent the carriage rate among healthy individuals, the pooled prevalence of MRSA in Ethiopia was found to be 32.5% [12].

The vulnerability of *S. aureus* to become resistant to antimicrobial drugs is one of the most urgent problems connected with it. *S. aureus* is a major agent within the antibiotic resistance approach since the 1960s, emerging immediately after the introduction of penicillin [13]. Almost all antimicrobial agents, including penicillins, cephalosporins, tetracyclines, chloramphenicol, methicillin, vancomycin, and sulphonamides, have been found to be notoriously resistant to *S. aureus*. The resistance level to vancomycin is particularly concerning [12]. *S. aureus*, one of the most prevalent microbes globally, has increasingly developed multidrug resistance, amplifying its overall burden worldwide. The impact of *S. aureus* encompasses prolonged hospital stays, long-term disabilities, heightened microbial resistance to antimicrobials, substantial financial strains on healthcare systems, elevated costs for patients and families, and increased mortality [14].

Patients infected with MRSA can develop severe complications such as sepsis, endocarditis, necrotizing pneumonia, orchitis, and osteomyelitis, leading to extended hospitalizations averaging approximately 40 days [15]. Globally, S. aureus significantly contributes to hospital admissions for pneumonia in young children [16]. Importantly, MRSA infections are linked to higher mortality rates and prolonged hospital stays compared to MSSA infections, thereby imposing a substantial economic burden [17]. Hence, the high prevalence of the strain, an increase in multidrug resistance, and being a primary cause of health burden throughout the world, *S. aureus* needs to be investigated more and more. Considering this, this study aimed to assess the carriage rate of *S. aureus* and its resistance pattern in Ethiopia.

To address the local context, this study specifically focuses on Ethiopia, where the epidemiology of *S. aureus* and MRSA is underexplored. Although previous studies primarily concentrated on hospitalized patients, the carriage rates and resistance patterns in healthy individuals and community settings remain largely unexamined, potentially revealing different prevalence and resistance dynamics. Additionally, while the global burden of *S. aureus* resistance is well-documented, the environmental, socioeconomic, and healthcare factors unique to Ethiopia, including access to healthcare, sanitation, and antibiotic use, likely play a significant role in influencing these rates. These gaps underscore the need for this study to provide a comprehensive analysis of *S. aureus* carriage and resistance in Ethiopia, offering valuable insights for public health policies and interventions aimed at controlling antimicrobial resistance in the region.

## Methods

### Protocol registration and reporting

The study adhered to the Preferred Reporting Items for Systematic Reviews and Meta-analysis (PRISMA) [18] guideline for synthesis and reporting of the findings. The protocol employed for this systematic review and meta-analysis has been registered in the Prospero database with the registration number PROSPERO 2024: CRD42023459698.

### Databases and search strategy

A comprehensive search was conducted in electronic databases including PubMed, Google Scholar, Hinari, SCOPUS, and EMBASE for articles published between 2013 and 2024. The search strategy was collaboratively developed by two study authors, and its execution was carried out by another two individuals. The search was conducted using MeSH terms, and combined key terms are taken from the review question. All potentially eligible studies were accessed by using the following combination keys; *S. aureus* * OR “ Staphylococcus aureus” OR SA OR “MSSA” OR “MRSA*” AND carriage rate” OR carriers OR AND Ethiopia. Endnote 20 software was used to manage references and remove duplicates. Despite extensive efforts to trace grey literature through various means, we found no relevant unpublished data.

### Inclusion and exclusion criteria

The search encompassed studies published before the search date. Inclusion criteria comprised studies meeting the following conditions: [1] investigations involving *S. aureus* carriage rate and/or reporting its antimicrobial susceptibility; [2] observational studies, encompassing cross-sectional studies, cohorts, or surveillance designs; [3] studies conducted in Ethiopia; [4] studies published in the English language. Exclusion criteria encompassed case reports, reviews, commentaries, and editorials. Additionally, conference abstracts were not included in the search (supplementary file 1 in S1 Data).

### Study selection and quality assessment

We utilized EndNote version 20.5 Reference Manager software [19], a tool designed for managing and organizing references and citations, to eliminate duplicate studies.A complete list of all studies retrieved, along with reasons for exclusion at the full-text review stage, is provided in S1 Table. The titles and abstracts were independently screened by two authors (MY and NA) to determine which articles should undergo a full-text review. The full text of the remaining articles was then obtained, and two investigators, TS and MH, independently assessed them for eligibility. The quality of the studies was evaluated using the JBI critical appraisal checklist [20]. The following criteria were utilized for appraising the selected studies: [I] Appropriateness of the sampling frame for addressing the target population, and the study participants’ sampling technique and adequacy of the sample size, [II] detailed description of study subjects and setting, [III] sufficient analysis of the data and validity reliability of methods used for measuring AMR and the carriage rate of *S. aureus*, [IV] appropriateness of the statistical analysis used and adequacy of the sample size. Disagreements were resolved through consensus and by involving a third reviewer. Studies scoring five and above out of nine JBI score points were considered to have a low risk. Our JBI checklist assessment found no studies classified as poor quality. The full appraisal results for each criterion across all included studies are presented in S2 Table.

### Data extraction

Data extraction was conducted by three authors (MY, and TS) using an established format in Microsoft Excel. To ensure accuracy, any discrepancies were resolved by repeating the procedure. MH and NA then compiled the articles that met our inclusion criteria into tables, summarizing information on authors, study period, publication year, study design, study setting, study population, study region, sample size, number of isolates, AMR prevalence for tested antimicrobials, and the antimicrobial sensitivity test methods employed. The final dataset was inspected and cleaned by three other authors (MY, GW and MB).

### Data analysis

To estimate the carriage rate of *S. aureus* and its AMR prevalence for tested antimicrobials, we employed a weighted inverse variance random-effects model [21]. We selected the random-effects model because we anticipated clinical and methodological heterogeneity among the included studies, such as differences in study settings, population characteristics, and diagnostic criteria. This model assumes that the true effect size varies between studies and thus provides a more conservative and generalizable estimate than the fixed-effect model. Subgroup analysis based on the region where the studies were conducted, as well as the age, occupation, HIV status, and study period (by considering the occurrence of COVID-19 as a means of dichotomization), were conducted to adjust for variation in pooled prevalence estimates. Heterogeneity among studies was evaluated using a forest plot, meta-regression, and the I^2^ statistic, where values of 25%, 50%, and 75% represented low, moderate, and high heterogeneity, respectively [22]. A Q test was used to determine the degree of heterogeneity, with a p-value less than 0.05 indicating significant heterogeneity. To assess publication bias, we employed a funnel plot and Egger’s regression test, where a p-value less than 0.05 suggested significant publication bias. Additionally, trim and fill analysis was applied to further evaluate the presence of publication bias in details. [23]. A sensitivity analysis was conducted to ensure the stability of the summary estimate. The meta-analysis was performed using STATA version 17 statistical software.

### Outcome of Interest

The study aimed to investigate two primary outcomes related to *S. aureus* in Ethiopia. Firstly, it focused on determining the nasopharyngeal carriage rate of *S. aureus* by calculating the proportion of *S. aureus* carriers among the total individuals tested, with prevalence data points pooled to estimate the overall carriage rate. *S. aureus* carriage studies defined a positive case as the detection of S. aureus from nasopharyngeal or nasal swab specimens using standard microbiological techniques, typically including culture on selective media (e.g., mannitol salt agar) followed by confirmatory biochemical tests (such as catalase and coagulase tests). Secondly, the study examined the antimicrobial resistance profiles of *S. aureus* against specific antimicrobials, including cotrimoxazole, clindamycin, erythromycin, penicillin, ampicillin, chloramphenicol, gentamycin, tetracycline, amikacin, ceftriaxone, ciprofloxacin, vancomycin, doxycycline, kanamycin, oxacillin, amoxicillin-clavulanic acid, and amoxicillin. To assess the pooled prevalence of antimicrobial resistance in *S. aureus* for these selected antimicrobials, all included primary studies adhered to the Clinical and Laboratory Standards Institute (CLSI) guidelines and employed the Kirby-Bauer disc diffusion method for antimicrobial sensitivity testing.

## Results

### Characteristics of included studies

**Fig 1:**
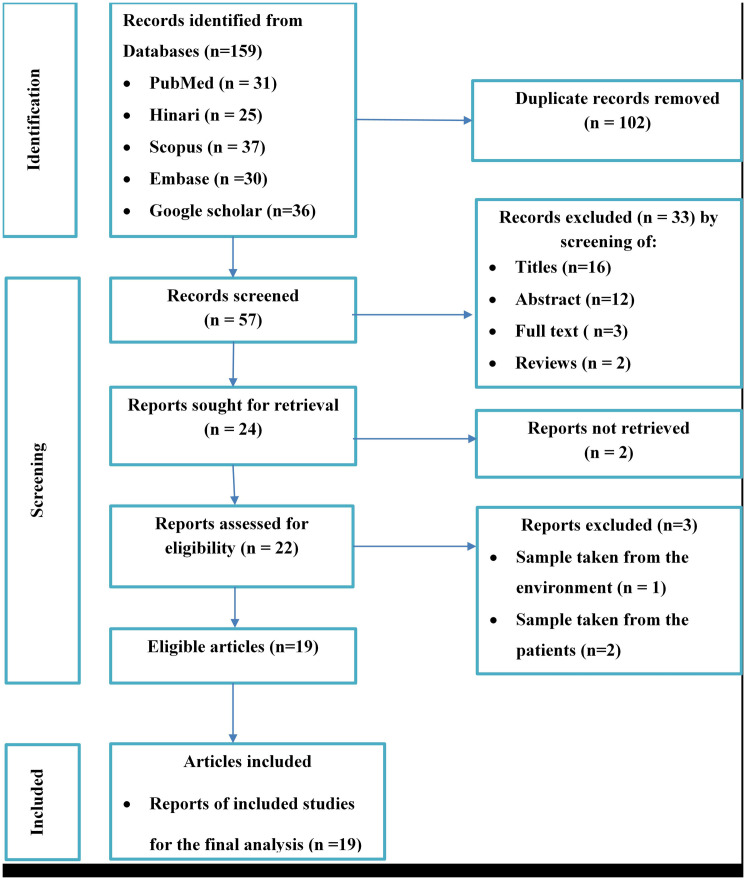
PRISMA flow diagram for the included studies along with reasons of exclusion for estimation of pooled carriage rate of *S. aureus* in Ethiopia.

A total of 159 potential studies were identified, comprising 31 articles from PubMed, 25 from Hinari (research4life), 30 from EMBASE, 37 from Scopus, and 36 from various other sources. The outcomes of the search and the reasons for exclusion during the study selection process are depicted in Fig 1. Ultimately, 19 articles were included to evaluate the carriage rate of *S. aureus* in Ethiopia. All the included studies adopted a cross-sectional study design, with eight of them specifically conducted in the Amhara region [24–31], three in Oromia [32–34], two in SNNPR [35,36], two in Addis Ababa [37,38], one in Harari [39] and three in Tigray [40–42]. Notably, not all the included studies did not report the antimicrobial susceptibility profile and the carriage rate of *S. aureus*. The study encompassed a total of 6798 participants, of which 1584 individuals were carriers of *S. aureus* and 1420 total isolates were tested to assess the antimicrobial resistance profile of *S. aureus*. Table 1 provides an overview of the characteristics of the included studies.

**Table 1:** Characteristics of studies included for the systematic review and meta-analysis of the prevalence of carriage rate of *S. aureus* in Ethiopia.

Author	Year of study	Region	City	Study population	Sample Size	Total No of SA Isolates	No of MRSA Isolates
Dagnew et al.	2011	Amhara	Gondar	Adult	200	41	4
Reta et al.	2013	Amhara	Bahir Dar	Pediatrics	300	123	17
Reta et al.	2015	Amhara	Debre Markos	Pediatrics	400	52	0
Kahsay et al.	2016	Tigray	Mekelle	Adult	384	69	24
Efa et al.	2016	Oromia	Jimma	Adult	371	82	31
Legesse et al.	2016	Tigray	Wukro & Adigrat	Adult	242	29	14
Beyene et al.	2017	Oromia	Jimma	Mixed	300	86	6
Manilal et al.	2017	SNNRP	Arba Minch	Adult	307	122	64
Tigabu et al.	2018	Amhara	Gondar	Pediatrics	622	143	14
Abie et al.	2019	Amhara	Gondar	Mixed	436	101	21
Mulu et al.	2020	Amhara	Gondar	Adult	423	60	8
Mekuriya et al.	2020	SNNRP	Arba Minch	Adult	258	70	19
Wonde et al.	2021	Harari	Harar	Adult	295	46	33
Kejela et al.	2010-2011	Oromia	Jimma	Mixed	354	169	39
Shibabaw et al.	2010-2011	Amhara	Dessie	Adult	118	34	15
Gebremedhin et al.	2014-2015	Tigray	Mekelle	Mixed	249	81	6
Mulu et al.	2016-2017	Amhara	Bahir Dar	Pediatrics	300	88	29
Gebre et al.	2016-2018	AA	AA	Pediatrics	183	50	5
Desta et al.	2018-2019	AA	AA	Adult	1056	138	29

SNNPR, southern nation nationalities and people region; AA, addis ababa; SA, *Staphylococcus aureus*; MRSA, methicillin-resistant *Staphylococcus aureus*.

### Quality of the included studies

All studies were evaluated using the JBI critical appraisal checklist for studies reporting prevalence data. Assessments conducted with the JBI quality appraisal checklists indicated that none of the included studies were considered to be of poor quality. Consequently, none of the studies were excluded from the meta-analysis. Although none of the included studies were excluded from the meta-analysis due to poor overall quality, several studies demonstrated notable weaknesses. Common limitations included lack of sample size calculation, absence of a clearly defined sampling frame, insufficient detail in statistical analysis, and in some cases, inadequate sample sizes. These issues may introduce potential selection bias, limit the representativeness of findings, or reduce the statistical power of individual studies.

### Meta‑analysis

#### Pooled carriage rate of *S. aureus* in Ethiopia.

**Fig 2:**
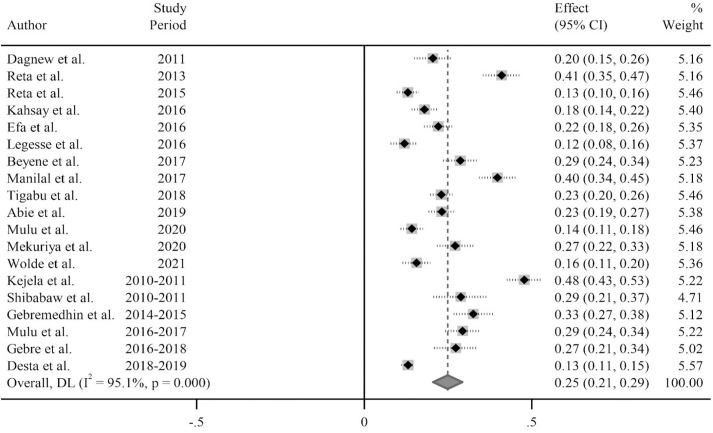
The pooled Carriage rate of S. aureus in Ethiopia.

In this systematic review and meta-analysis conducted in Ethiopia, the estimated pooled carriage rate of *S. aureus* was 25% (95% CI: 21–29%). The heterogeneity test showed an I^2^ value of 95.4%, indicating significant variation among the included studies (p < 0.001) (Fig 2).

#### The pooled odds ratio of MRSA and MSSA.

**Fig 3:**
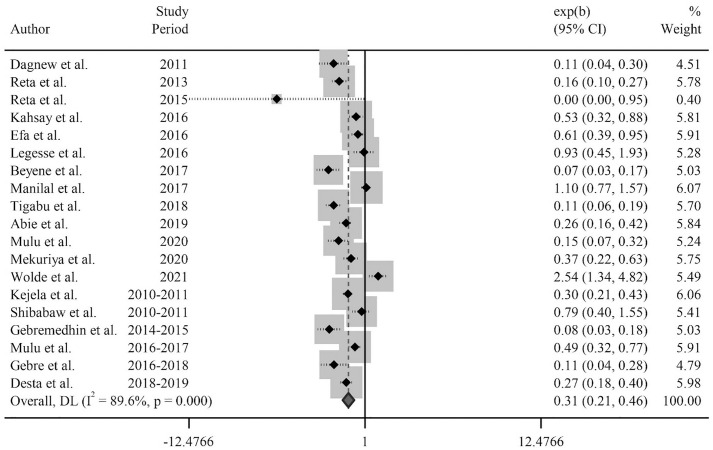
Pooled estimate of the odds ratio between MRSA and MSSA from the carriage isolates of *S. aureus* in Ethiopia.

The estimated pooled odds ratio between MRSA and MSSA from the identified carriage isolates of *S. aureus* in Ethiopia stood at 0.31, with a 95% CI spanning from 0.21 to 0.46. (I^2 ^= 89.6%, p < 0.001) (Fig 3).

### Subgroup analysis

The subgroup analysis, divided by year of study, regions, age groups, HIV status, and job roles of participants, revealed diverse pooled rates of *S. aureus* carriage. To enhance generalizability by accounting for regional variations, temporal trends, and contextual factors such as antibiotic policies, we performed subgroup analyses based on these key factors. Among these findings, the highest pooled *S. aureu*s carriage rates were observed in studies conducted before 2019 (26%, 95% CI 21, 31), in the Oromia (33%, 95% CI 18, 48) and SNNPR regions (33%, 95% CI 21, 46), in studies assessing mixed age groups [involving both pediatric and adult groups] (33%, 95% CI 22, 44), among HIV positive patients (32%, 95% CI 27, 38), and among participants with undefined job roles (30%, 95% CI 22, 38), as delineated in Table 2.

**Table 2:** Summary of subgroup analysis detailing the carriage rate of S. aureus across various subgroups.

Groups	Subgroups	Pooled estimate of carriage rate (95% CI)	I^2^(%)	P-value
Year	Before 2019	26(21,31)	95.6	< 0.001
	After 2019	19(12,26)	87.9	< 0.001
Age	Adult	21(16,25)	92.4	< 0.001
	Pediatrics	27(18,36)	95.2	< 0.001
	Mixed	33(22,44)	94.6	< 0.001
Region	Amhara	24(18,30)	93.3	< 0.001
	Addis Ababa	20(6,34)	94.1	< 0.001
	Oromia	33(18,48)	96.5	< 0.001
	SNNPR	33(21,46)	90.3	0.002
	Tigray	21(10,31)	93.8	< 0.001
HIV status	Unknown	23(18,28)	95.1	< 0.001
	Positive	32(27,38)	71.8	0.014
Job	Unknown	30(22,38)	96.6	< 0.001
	Food handler	25(17,33)	77.6	0.035
	Janitors	21(15,26)	70.7	0.065
	Health care workers	19(14,24)	89	< 0.001

### Heterogeneity analysis

The studies included in the analysis exhibited substantial heterogeneity (I^2^ = 95.1%; p-value < 0.001), which was not adequately addressed by a weighted inverse variance random-effects model. To further explore this heterogeneity, we employed a forest plot (Fig 2) for a subjective assessment and carried out a subgroup analysis. To further assess the sources of heterogeneity, meta-regression was conducted using sample size and study periods. The analysis revealed that neither of these factors contributed significantly to the observed heterogeneity (Table 3).

**Table 3:** Meta-regression of carriage rate of S. aureus with sample size and year of publication.

P	Coefficient	Std. err.	t	P > |t|	[95% conf. interval]	
**Sample Size**	−0.000166	0.000114	−1.5	0.162	−.0004051	.0000737
**_cons**	0.3091439	0.046974	6.58	0.001	.2100377	.40825
**year**	−0.013837	0.00676	−2.1	0.056	−.0280991	.0004247
**_cons**	28.17585	13.64303	2.07	0.054	−.6084141	56.96012

### Publication bias

**Fig 4:**
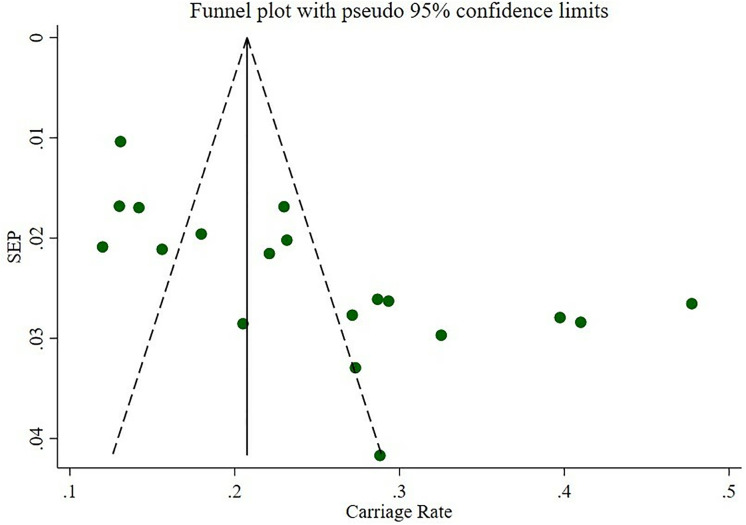
Funnel plot of carriage rate of *S. aureus* in Ethiopia.

Procedurally, we first evaluated publication bias through a subjective examination of the funnel plot (Fig 4), which revealed an asymmetrical distribution of *S. aureus* carriage rates around the pooled estimate, suggesting potential bias. To further assess this, we conducted Egger’s regression test, which yielded a p-value < 0.001, confirming the presence of publication bias. we acknowledge that these methods have certain limitations. For example, funnel plot asymmetry can be influenced not only by publication bias but also by true heterogeneity, small study effects, or methodological differences across studies. Similarly, Egger’s test may have limited power when the number of studies is small, potentially leading to false-negative results. Additionally, a trim and fill analysis was performed to account for overlooked small studies, which added eight studies to the analysis, further indicating the presence of such biases.

### Trim and fill analysis

To evaluate the influence of overlooked studies on the carriage of *S. aureus* in Ethiopia a trim and fill analysis was performed. The results of trim and fill analysis revealed there were eight added overlooked studies, which recalibrated the pooled carriage rate to 17.4% (95% CI: 12.6, 22.2).

### Sensitivity Analysis

We conducted a sensitivity analysis for the carriage rate of *S. aureus* using a random effects model. we conducted leave-one-out analyses to assess the influence of each individual study on the overall pooled estimates. This approach allowed us to identify whether any single study had a disproportionate effect on the results. Excluding each study individually did not significantly affect the estimated carriage rate of *S. aureus* in Ethiopia. This indicates the absence of significant outliers among the included studies and confirms the robustness of our findings.

### Antimicrobial resistance profiles of *S. aureus* in Ethiopia

We systematically collected data on the sensitivity of *S. aureus* to seventeen antimicrobials and conducted an analysis to determine the pooled estimate of resistance. Our study findings revealed that vancomycin exhibited the highest efficacy at 3% (95% CI 0, 5), followed by clindamycin at 6% (95% CI 4, 8). In contrast, penicillin emerged as the least effective drug with a resistance rate of 93% (95% CI 91, 96), followed by ampicillin at 80% (95% CI 69, 90), and oxacillin at 49% (95% CI 1, 97). Notably, moderate levels of resistance were observed in macrolides (erythromycin 23%), aminoglycoside (gentamicin 12% and quinolone (ciprofloxacin 12%), as illustrated in Table 4.

**Table 4:** Pooled estimates of antimicrobial resistance profile of *S. aureus* in Ethiopia.

Antimicrobials	Number of isolates tested	Pooled Estimates of AMR (95% CI)	I^2^ (%)	P-value
Co-trimoxazole	1457	29(20,38)	96	< 0.001
Clindamycin	1094	6(4,8)	84.3	< 0.001
Erythromycin	1561	23(18,28)	96.3	< 0.001
Penicillin	1143	93(91,96)	87.4	< 0.001
Ampicillin	481	80(69,90)	91.7	< 0.001
Chloramphenicol	907	18(10,27)	97	< 0.001
Tetracycline	1266	43(33,52)	93.2	< 0.001
Gentamycin	875	12(8,16)	92.6	< 0.001
Ciprofloxacin	1031	12(8,16)	89.9	< 0.001
Vancomycin	394	3(0,5)	82.8	< 0.001
Doxycycline	262	21(15,27)	39.3	0.159
Oxacillin	235	49(1,97)	99.8	< 0.001
Amoxicillin -Clavulanic acid	138	14(3,30)	86.4	0.007

AMR, antimicrobial resistance.

## Discussion

This systematic review and meta-analysis is a pioneering study that thoroughly investigates the pooled carriage rate of *S. aureus* along with its antimicrobial susceptibility profile within the Ethiopian healthcare context. Our analysis estimated the pooled carriage rate of *S. aureus* in Ethiopia to be 25% (95% CI: 21−29), with the MRSA carriage rate at 7% (95% CI: 4−10). Additionally, the estimated pooled odds ratio between MRSA and MSSA among the identified *S. aureus* carriage isolates in Ethiopia was 0.31 (95% CI: 0.21–0.46). These findings are relatively lower compared to a previous systematic review and meta-analysis, which reported an overall pooled prevalence of *S. aureus* and MRSA colonization at 30.9% and 10.94%, respectively [43]. Poor hand hygiene practice, presence of limited infrastructure, inadequate knowledge, lack of personal protective equipment, inappropriate and overuse of antibiotics, absence of good microbiological laboratory capacity for microbiological isolation and detection, and ineffective infection control measures could be possible reasons for the reported higher carriage rate of *S. aureus* in Ethiopia.

Our finding is in line with a systematic review and meta-analysis conducted in Iran on *S. aureus* nasal carriage and MRSA among medical students that showed a 28% pooled prevalence of nasal *S. aureus* carriage and 2% MRSA [44]. Likewise, another meta-analysis [45] done in similar setting on nasal carriage rates among healthcare workers (HCWs) showed the prevalence of *S. aureus* to be 22.7%. Consistent with our finding, a meta-analysis study conducted by Albrich *et al*. found the *S. aureus* and MRSA prevalence among HCWs to be 23.7% and 4.6%, respectively [46]. The pooled carriage rate of MRSA in this meta-analysis is lower than a meta-analysis done in Iran [45] and Egypt [47], revealing 32.8% and 32% MRSA prevalence, and higher than a meta-analysis done in Europe and the United States of America, displaying 1.8% carriage rate. Contrary to our finding, a systematic review extracted mostly from high income countries spotlighted a 2.1%, 9.5%, and 16.6% proportion of MRSA carriage among neonate mothers, healthcare workers, and environmental samples, respectively [48]. Similarly, a systematic review and meta-analysis [49] done in Asia-Pacific region from 2000–2016 displayed a community-associated MRSA carriage prevalence of 0% to 23.5% in the general public and 0.7% to 10.4% in hospital setup. The noticeable differences observed in MRSA carriage rate between countries is a sum effect of both methodological and infection control policy differences. For instance, variations in sampling techniques, healthcare access, cultural norms, sample size, microbiological isolation and detection techniques, and sampling sites may contribute to the discrepancy. Overall, the present meta-analysis finding highlighted that early identification of carrier rate of *S. aureus* and subsequent reduction of this pathogen is helpful in reducing transmission and controlling the spread of MRSA infections.

The sub-group analysis done based on the specific regions of the country unveiled that Oromia and SNNPR regions exhibited the highest *S. aureus* carriage at a similar rate of 33% while the capital city (Addis Ababa) demonstrated the least carriage rate at 20%. Although the precise numerical figure varies, this finding is congruent to a previous meta-analysis [43] which unveiled highest *S. aureus* and MRSA prevalence (47.74%, 21.28%) of nasal colonization from Oromia region. Unlike our subgroup analysis finding demonstrating humblest *S. aureus* carriage rate in Addis Ababa, previous systematic review and meta-analysis reported lowest prevalence of nasal colonization of *S. aureus* and MRSA (20.83%, 4.82%) in Tigray region [43]. The discrepancy could be ascribed to variation in culture, norm, healthcare access, health infrastructure and health policy implementation across the regions of the country. A further subgroup analysis conducted on the basis of study years, age groups, HIV status, and participants job roles detected most prominent pooled *S. aureus* carriage rates amidst studies done before year 2019 (26%), studies involving both adult and pediatric age groups (33%), in HIV positive patients (32%), and participants with undefined job roles (30%). Despite our finding revealing most eminent *S. aureus* carriage in subjects with undefined jobs, previous systematic review and meta-analysis [44–50] reported substantial carriage rate of *S. aureus* and MRSA from health care workers. This fact could be best explained by poor hand hygiene knowledge and compliance, healthcare access, inadequate protective equipment, misuse of antibiotics, poor microbiological laboratory capacity and pitiful infection control policies.

The findings from our systematic review and meta-analysis reveal significant resistance patterns of *S. aureus* to various antimicrobials in Ethiopia. Notably, the resistance rates to commonly used antibiotics such as penicillin (93%), ampicillin (80%), tetracycline (43%), and oxacillin (48%) are alarmingly high. This high prevalence of resistance, especially to penicillin and ampicillin, suggests that these antibiotics may no longer be effective for treating *S. aureus* infections in the region. The substantial resistance to tetracycline and oxacillin indicates the presence of MRSA strains, which complicates treatment protocols due to their limited susceptibility to other antibiotics. Additionally, the resistance to other commonly prescribed antibiotics like doxycycline (21%) further limits the therapeutic options available to healthcare providers. Similarly, a meta-analysis of 98 studies done on the prevalence of resistance of *S. aureus* to different antibiotics in Nigeria indicated very high degree of resistance to penicillin G (82%), cloxacillin (77%), amoxicillin, cefuroxime (69%), and ampicillin (68%) and low degree of resistance to vancomycin (13%). The clinical implications of these findings are profound. The high resistance rates necessitate a reconsideration of empirical antibiotic therapy for *S. aureus* infections in Ethiopia. Healthcare providers need to be aware of these resistance patterns to avoid the use of ineffective antibiotics, which could lead to treatment failures and increased morbidity and mortality. Moreover, the low resistance rates to antibiotics such as clindamycin (6%), and vancomycin (3%) suggest that these antibiotics could be more effective options for treating *S. aureus* infections. However, the continued use of these antibiotics should be monitored closely to prevent the development of resistance. The data underscores the importance of ongoing surveillance and the implementation of stringent antibiotic stewardship programs to mitigate the spread of resistant *S. aureus* strains and preserve the efficacy of available antibiotics*.* These include developing national guidelines for antibiotic use, providing targeted training for healthcare providers, strengthening antimicrobial resistance surveillance, promoting diagnostic stewardship with rapid tests, launching public awareness campaigns on proper antibiotic use, and enhancing the role of pharmacists in antimicrobial stewardship.

The findings of our study reveal important insights into the carriage rate and antimicrobial resistance of *S. aureus* in Ethiopia. Despite substantial heterogeneity among the included studies (I^2^ = 95.1%; p < 0.001), our subgroup with various variables and meta-regression analysis by using sample size and study periods did not identify as significant sources of the observed variability. This suggests other unexamined factors may contribute to the observed heterogeneity. Publication bias was evident, as indicated by Egger’s regression test (p < 0.001), and supported by the trim and fill analysis, which added eight studies, adjusting the pooled carriage rate to 17.4% (95% CI: 12.6–22.2). The sensitivity analysis confirmed the robustness of our findings, showing that excluding individual studies did not significantly impact the carriage rate estimate. These results underscore the importance of accounting for potential biases and variability in meta-analyses and highlight the need for ongoing surveillance to better understand and manage *S. aureus* colonization and resistance patterns in Ethiopia. To effectively address the evolving issue of antimicrobial resistance in Ethiopia, future efforts should include long-term surveillance studies, investigations into the mechanisms of resistance, and targeted interventions for high-risk populations, such as healthcare workers and individuals living with HIV.

### Strengths and limitations of the study

This comprehensive systematic review and meta-analysis provide valuable insights into the carriage rate of *S. aureus* and MRSA in Ethiopia, along with their antimicrobial resistance profiles. These findings could inform better empirical management of infections caused by this microorganism and give emphasis on the application of better infection prevention strategies. However, the study has several limitations. Notably, confounding variables such as underlying health conditions, antibiotic use history were not addressed in the included primary studies. the representativeness of the included studies may be limited, as many were conducted in specific geographic regions or urban healthcare settings, with fewer studies representing rural or underserved populations There was also significant heterogeneity among the included studies, as evidenced by a high I^2^ statistic. Additionally, the primary studies predominantly used disc diffusion methods for antimicrobial sensitivity testing, which may not be as accurate as other methods like broth microdilution, gradient diffusion method, agar dilution, and colorimetric or fluorometric assays. Furthermore, for some antimicrobials, the number of isolates tested was limited, potentially undermining the reliability of the reported resistance rates. These limitations suggest that while the findings are informative, they should be interpreted with caution.

### Recommendation

We emphasize the need for targeted epidemiological studies that include both rural and underserved populations to improve representativeness and generalizability. We also recommend the implementation of long-term, nationwide antimicrobial resistance surveillance programs to monitor trends over time and guide policy decisions. In addition, we highlight the importance of interventional studies to evaluate the effectiveness of infection prevention strategies, antimicrobial stewardship interventions, and public health education campaigns tailored to the local context.

## Conclusion

The *S. aureus* and MRSA carriage rates in Ethiopia are consistent with many international studies but show regional variability. In Ethiopian healthcare settings, colonized *S. aureus* exhibits significant resistance to many commonly used antimicrobials. Therefore, urgent interventions are needed, including effective screening, robust infection control strategies, stringent disinfection procedures, and appropriate decolonization programs. These measures should be implemented regionally to prevent the transmission of MRSA and reduce the incidence of subsequent infections.

## Supporting information

S1 Table Complete List of Articles Identified in the Literature Search. (XLSX)

S2 Table JBI Critical Appraisal Checklist Scores for Included Studies. (DOCX)

S1 Data Supplementary File 1. (DOCX)

## Abbreviations

*S. aureus*: *Staphylococcus aureus*
MRSA: methicillin-resistant *S. aureus*
MSSA: methicillin-sensitive *S. aureus*
AMR: antimicrobial resistance
PRISMA: preferred reporting items for systematic reviews and meta-analysis
JBI: joanna briggs institute
CLSI: clinical and laboratory standards institute.
