# Supplementary material for: Carriage rates and antimicrobial resistance of Staphylococcus aureus and methicillin-resistant Staphylococcus aureus in Ethiopia: A systematic review and meta-analysis
Source: PLoS One. 2025 Oct 10;20(10):e0333054. doi: 10.1371/journal.pone.0333054 (PMC12513613; doi:10.1371/journal.pone.0333054)
Supplement: S2 Table — (DOCX) [file pone.0333054.s002.docx]

| **Author** | **year** | **Q1** | **Q2** | **Q3** | **Q4** | **Q5** | **Q6** | **Q7** | **Q8** | **Q9** | **Total**  **Score** | **Status** |
| --- | --- | --- | --- | --- | --- | --- | --- | --- | --- | --- | --- | --- |
| Abie et al. | 2020 | **Y** | **Y** | **Y** | **Y** | **Y** | **Y** | **U** | **Y** | **Y** | **8/9** | **Included** |
| Beyene et al. | 2019 | **Y** | **U** | **N** | **Y** | **Y** | **Y** | **N** | **Y** | **Y** | **6/9** | **Included** |
| Dagnew et al. | 2012 | **Y** | **Y** | **Y** | **Y** | **Y** | **U** | **U** | **Y** | **Y** | **7/9** | **Included** |
| Desta et al. | 2022 | **Y** | **Y** | **Y** | **Y** | **Y** | **U** | **U** | **Y** | **Y** | **7/9** | **Included** |
| Efa et al. | 2019 | **Y** | **U** | **U** | **Y** | **Y** | **Y** | **Y** | **Y** | **Y** | **7/9** | **Included** |
| Gebre et al. | 2023 | **Y** | **Y** | **Y** | **Y** | **Y** | **U** | **U** | **Y** | **Y** | **7/9** | **Included** |
| Gebremedhin et al. | 2016 | **Y** | **Y** | **Y** | **Y** | **Y** | **U** | **U** | **Y** | **Y** | **7/9** | **Included** |
| Kahsay et al. | 2018 | **Y** | **Y** | **Y** | **Y** | **Y** | **N** | **N** | **Y** | **Y** | **7/9** | **Included** |
| Kejela et al. | 2013 | **Y** | **Y** | **Y** | **Y** | **Y** | **N** | **N** | **Y** | **Y** | **7/9** | **Included** |
| Legesse et al. | 2018 | **U** | **U** | **Y** | **Y** | **Y** | **N** | **N** | **Y** | **Y** | **5/9** | **Included** |
| Manilal et al. | 2019 | **Y** | **Y** | **Y** | **Y** | **Y** | **N** | **U** | **Y** | **Y** | **7/9** | **Included** |
| Mekuriya et al. | 2022 | **N** | **Y** | **Y** | **N** | **N** | **Y** | **Y** | **Y** | **Y** | **6/9** | **Included** |
| Muhaba et al. | 2022 | **Y** | **Y** | **Y** | **Y** | **Y** | **Y** | **U** | **Y** | **NA** | **7/8** | **Included** |
| Mulu et al. | 2018 | **Y** | **Y** | **Y** | **Y** | **Y** | **Y** | **N** | **Y** | **Y** | **8/9** | **Included** |
| Mulu et al. | 2021 | **Y** | **N** | **Y** | **Y** | **Y** | **Y** | **Y** | **Y** | **N** | **7/9** | **Included** |
| Reta et al. | 2014 | **Y** | **Y** | **Y** | **Y** | **Y** | **Y** | **U** | **Y** | **Y** | **8/9** | **Included** |
| Reta et al. | 2017 | **Y** | **Y** | **Y** | **Y** | **Y** | **Y** | **U** | **Y** | **Y** | **8/9** | **Included** |
| Shibabaw et al. | 2014 | **Y** | **Y** | **Y** | **Y** | **N** | **Y** | **Y** | **N** | **Y** | **7/9** | **Included** |
| Tigabu et al. | 2018 | **Y** | **Y** | **Y** | **Y** | **Y** | **Y** | **Y** | **Y** | **Y** | **9/9** | **Included** |
| Wolde et al. | 2023 | **U** | **Y** | **Y** | **Y** | **Y** | **Y** | **Y** | **Y** | **Y** | **8/9** | **Included** |

**Table Quality assessment of studies included for meta-analysis Using JBI Checklist**

***Y: Yes, N:No,U:Unknown,NA:Not Applicable***
